# Dural repair after intraoperative CSF leakage in endoscopic endonasal skull base surgery without pedicled nasoseptal flap: is it a safe surgical technique?

**DOI:** 10.1007/s10143-025-03831-4

**Published:** 2025-09-29

**Authors:** Bernardo Reyes Medina, Stefan Linsler, Safwan Saffour, Kerim Hakan Sitoci-Ficici, Joachim Oertel

**Affiliations:** 1 Medical Faculty, Saarland University, Homburg, Germany; 2 Department of Neurosurgery, Hospital Bayreuth and Medical Campus Oberfranken of Friedrich Alexander University, Bayreuth, Germany; 3 Klinik für Neurochirurgie, Universität des Saarlandes Kirrbergerstrasse, Gebäude 90.5, 66424 Homburg, Germany

**Keywords:** Endoscopy, Multilayer closure, Endonasal transsphenoidal approach, Sellar pathology, CSF leakage, Skull base reconstruction

## Abstract

In this study we evaluated the outcome and efficacy of a multilayer closure technique of the sellar floor after transsphenoidal endoscopic approaches for sellar pathologies in correlation to the intraoperative grading of cerebrospinal fluid (CSF) leak. Overall 280 patients were reviewed retrospectively who underwent endonasal transsphenoidal endoscopic surgery for sellar pathologies from January 2011 to April 2020. Among them, 87 patients had an intraoperative cerebrospinal fluid leak and were included in this study. The intraoperative cerebrospinal fluid grading was based on the classification by Esposito. Clinical outcomes were evaluated based on postoperative complications and development of postoperative cerebrospinal fluid leak in correlation to the used closure technique and material of dural and skull base reconstruction. Their association with the intraoperative cerebrospinal fluid leak grade, underlying pathology and the tumor size were analyzed as well. From the 87 patients, there was 54 women (62%) and 33 men (38%). The mean age was 56.3 ± 14.8. The most frequent histological diagnosis was non-secreting adenoma in 40 cases (45%), followed by the secreting adenomas with 16 cases (18%). Eight cases of meningioma (9%), six cases of Rathke’s cleft cyst (7%), six cases of craniopharyngioma (7%), four cases of colloid cysts (4%), four chordomas (4%) were observed. Of the 87 patients with intraoperative cerebrospinal fluid leaks, 20 cases were classified as grade 1, 37 cases as grade 2 and 30 cases as grade 3. The materials used for the closure of grade 1 were primarily sealant sponge Tachosil ® (70%), followed by fibrin glue (55%) and bone (55%). For grade 2, Tachosil ® (78%), bone (57%) and autologous fat graft (54%). In grade 3 dural leaks, the combination of lumbar drains (90%), autologous fat graft (87%) and fascia lata (60%) were mainly used. Only nine patients developed a new postoperative cerebrospinal fluid leak in this series, and were treated conservatively with lumbar drains, but only three of them discontinued by this method. The remaining six patients with persistent postoperative cerebrospinal fluid leak underwent revision surgery. The analyzed multilayer closure technique correlated to the intraoperative CSF leakage for endoscopic transsphenoidal surgery has been shown to be safe. This study shows a lower intraoperative cerebrospinal fluid leak rate compared to other studies and similar rate of postoperative cerebrospinal fluid leak (3.1% vs. 3.9%) compared to the pedicled vascular flap and other techniques in the literature. Based on these results, the multilayer closure represents a real alternative to nasoseptal flap application in selected cases. However, in large defects and complex reconstruction of anterior skull base defects, use of lumbar drain and pedicled nasoseptal flap should remain the gold standard of treatment.

**Supplementary Information:**

The online version contains supplementary material available at 10.1007/s10143-025-03831-4.

## Introduction

The use of the endoscope in the transsphenoidal approach was first introduced in the late 1970 s and was mostly used as technical help to the use of a microscope [1]. Cappabianca was a pioneer in the use of the pure endoscopic transsphenoidal approach [2] parallelly to Jho and Carrau [3]. In the last years, the transsphenoidal approach has been used not only to treat sellar pathologies but also in extrasellar pathologies, due to the further refinement of neurosurgical techniques [4–7].

Thereby, dural closure and skull base reconstruction have remained one of the main challenges in the resection of large tumors and different intradural pathologies via endonasal transsphenoidal approach [8]. The risk of postoperative CSF leakage (2–10%), meningitis (0.6–1.5%), epistaxis (3%) as well as other postoperative correlated complications remain relatively high and avoidance of these complications is a central issue for the surgeons [9–16]. Many reconstruction techniques have been proposed to repair the sellar floor, and numerous materials can be used, such as fat, muscle, fascia lata, vascularized mucosal flaps, vicryl patches, bone, absorbable and non-absorbable plates, titanium mesh plates, tissue sealants, and use of lumbar drains [17–29].

The selected technique for dural closure highly depends on the structure of the dural defects and the personal preference and experience of the performing surgeon. A classification of the intraoperative CSF leaks has been described by Esposito, and according to its grade, the adequate repair of the sellar floor, consisting in grade 0 to grade 3 [17].

A technique called nasoseptal flap or Haddad flap has been described by Hadad et al. in 2006 [18]. This technique is the most famous and the most widely used technique in the repair of the sellar floor after sellar transsphenoidal surgery. It uses a neurovascular pedicled flap of the nasal septum mucoperiosteum and mucoperichondrium based on the nasoseptal artery (branch of the posterior septal artery and the terminal branch of the internal maxillary artery) and harvested carefully according to the size of the sellar floor defect [18, 30, 31]. Additionally, a collagen matrix is used as inlay graft, abdominal fat or onlay fascial graft, and the use of fibrin glue can be helpful to secure the flap [18, 26, 27, 32–37]. The nasoseptal flap has significantly reduced the incidence of postoperative CSF leaks [30] and has become one of the most effective techniques in the sellar floor reconstruction with CSF leakage. However, its use requires meticulous preparation in order to preserve the integrity of the flap, resulting in longer surgery times. Additionally, nasal complaints and complications are described in literature in detail [11]. Therefore, the authors preferred a surgical technique without nasoseptal flap preparation and different skull base reconstruction to reduce the surgical trauma in the nostrils.

This study analyzes the outcomes of the closure technique applied in our neurosurgical department in case of endoscopic transsphenoidal surgery for sellar pathologies. The main goal of this study is to analyze the effectiveness of the presented closure technique and rate of postoperative CSF leakages compared to the literature and the current dural closure techniques.

## Materials and methods

### Patient criteria

The data of all patients were retrospectively reviewed from January 2011 and April 2020, who presented with intrasellar pathologies and underwent transsphenoidal endoscopic surgery in the department of neurosurgery at University hospital of Saarland, Homburg/Saar, Germany. The study was performed applying ethical standard according to the Declaration of Helsinki. Ethical approval for this study was obtained from the ethical committee of the Ärztekammer des Saarlandes (trial number: 44/21).

The inclusion criteria were: surgery via a transsphenoidal endoscopic approach for sellar pathology, intra- and/or postoperative identification of a CSF leak based on the surgical videos and the follow up and complete pre- and postoperative ophthalmological, endocrinological and radiological evaluation and follow up. The exclusion criteria were: patients younger than 18 years and patients with traumatic CSF leakage, incomplete data sets. All surgeries were conducted by two experienced neurosurgeons (JO, SL).

A total of 280 patients were analyzed primarily. Of those 280 patients, 87 patients presented an intraoperative CSF flow on the surgical videos. The study cohort is exclusively composed of these 87 patients. No postoperative CSF fistula was noted in the 193 patients without intraoperative CSF leakage.

For data collection, all available documents were reviewed including medical records, surgical and histopathological reports, video recordings, clinical visits and pre- and postoperative imaging studies. The retrospective study was authorized by the ethical committee of the medical association of the Saarland (No 42/21).

### Perioperative management

All patients with peri- and intrasellar lesions underwent visual function evaluations including formal visual field testing. The postoperative visits were performed during the patients’ in-hospital stay within the first week after surgery and six weeks after surgery followed by a variable time schedule depending on their hormonal and magnetic resonance imaging (MRI) findings. Preoperative and postoperative MRI was obtained routinely. A routine preoperative computer tomographic scan with axial and coronal reformations was performed to define the bony boundaries of the sellar region including the sphenoid cavity. If an MRI was not applicable, only a computertomography was performed. All patients with pituitary adenomas received perioperatively a stress dose of hydrocortisone 100 mg/24 hours as standard. After surgery, all patients were kept overnight at the intermediate care unit. If there was an intraoperative severe rhinoliquorrhoea, patients were treated with lumbar drainage routinely for 5 days and antibiotic prophylaxis (second generation cephalosporin) during this time period [6, 19, 38]. Please also refer to the results section for further explanation.

### Surgical management

All surgeries were performed under general anaesthesia with orotracheal intubation. Although there is a close cooperation with the ENT department, only cases with a combined transcranial and endonasal approach were performed together. None of these patients was included in this study. The surgeries of this study were performed by the authors without ENT surgeon.

The patient was maintained in a supine position with the upper part of the body slightly elevated (about 20°) and the head tilted towards the left. The patient’s head was fixed with a three-pin head-fixation system. Lateral fluoroscopy (C-arm) was routinely used for intraoperative imaging. MRI- or CT-based neuronavigation was administered. The nasal cavities were prepared with an alcohol-based disinfectant. Mepivacaine with 1:100 000 adrenaline-soaked cotton was placed into bilateral nasal cavities for local haemostasis. The periumbilical abdomen and right thigh were also prepared for fat and fascia lata graft harvesting. The patient, the C-arm, and the endoscopic equipment were sterile draped.

The endoscopic equipment consists of a series of variously angulated rigid-rod lenses Hopkins optics, a Xenon cold light source, a digital one-chip camera, a high-resolution video monitor screen and a digital recording system. For surgery, 4 mm–2.7 mm rigid endoscopes with Hopkins optics and 0°-angled lenses were used for the approach and subsequent tumor removal. Scopes with 30°- and 45°-angled lenses were employed for final inspection to improve radicality as well as for tumor resection; this was especially useful if “a look around the corner” was required such as in tumors located far laterally, in the cavernous sinus or in the suprasellar region. All equipment was provided by Karl Storz Company, Tuttlingen, Germany [6, 19, 39].

For wound closure the following technique was applied: If the sellar diaphragm did not descent in tumours with suprasellar extension, a Valsalva manoeuvre was performed to bring residual tumour tissue and the diaphragm down into sight. At the end of the procedure, the 0° optics were removed and angled optics inserted. In case of observed CSF leakage, closure of the sella was performed with autologous fat, fascia lata, sealant sponge (Tachosil^®^), fibrin glue, bone and lumbar drains. Up to this study, there was no protocol of the use of the different closure materials. The choise of the different materials in every single case was based on the intraoperative identification of cerebrospinal fluid leak and the surgeon´s experience. In cases with almost undamaged stable diaphragm, the diaphragm was reconstructed with Tachosil sponge. The sellar floor was reconstructed with bone pieces and – if indicated – the sphenoid cavity was partially filled with an additional fat/gelfoam graft. The sphenoid floor was reconstructed with bone sampled from the approach (see exemplary suppl. video 1 and suppl. video 2). All procedures were video recorded. The dural defect was analysed and classified according to the classification introduced by Esposito et al. [17]: grade 0 shows no cerebrospinal fluid leak, grade 1 consists of a small cerebrospinal leak confirmed with Valsalva manoeuvre, grade 2 consists in a moderate cerebrospinal leak with obvious diaphragmatic defect, and grade 3 shows a large cerebrospinal leak with large diaphragmatic defect.

### Statistics

The illustrations and analysis of data were performed using SPSS (SPSS, version 22, IBM Corporation, NY, US). Collected data were compared using Pearson correlation as Mann-Whitney-U-test to compare differences. Significance level was set at *p* < 0.05. Values are presented as means ± standard deviation.

## Results

### General results

Of the 87 patients presenting an intraoperative CSF leak, fifty-four patients (62%) were female and 33 (38%) male. The mean age was 56.3 ± 14.8, range 22–84 years. The most frequent histological diagnosis was non-secreting adenoma in 40 cases (45%). Secreting adenomas were the second most common diagnosis with 16 cases (18%), in which seven cases of adrenocorticotropic hormone secreting adenoma, five cases of prolactinoma and four cases of growth hormone releasing adenoma were observed. Eight cases of meningioma were diagnosed (9%), six cases of Rathke’s cleft cyst (7%), six cases of craniopharyngioma (7%), four cases of colloid cysts (4%), four chordomas (4%) and there was only one single case each of cavernous hemangioma, arachnoid cyst and metastatic lesion respectively.

The mean volume of tumors was 4.99 cm^3^ *±* 0.69. The mean surgical time scored 106 min + 11.4. Mean follow-up was 56 + 13.8 months.

### Surgical outcome

Out of the 87 cases with intraoperative CSF leakage, postoperative CSF leaks were found in nine cases (10%), meningitis was observed in five cases (5,7%). A postoperative hemorrhage occurred in five cases (5,7%), a transient diabetes insipidus in eight cases (9%), a permanent diabetes insipidus in four cases (4,5%) and persisting nasal adhesions requiring ENT treatment in five cases (5,7%). No mortality occurred.

### Intraoperative CSF leakage

**Fig. 1:**
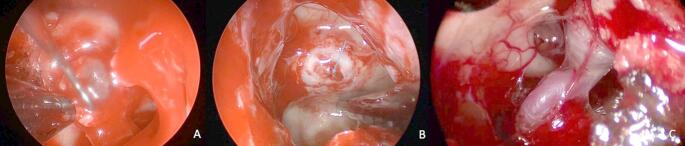
Exemplary intraoperative image of a CSF leakage (**A**) with CSF flow without obvious diaphragmatic defect (grade 1), (**B**) moderate leak with small diaphragmatic defect (grade 2) and (**C**) large dural defect in case of intradural pathology (grade 3)

The intraoperative CSF leaks were graded using the classification by Esposito (see Table 1). The most encountered CSF leak was grade 2 (*n* = 37, 42.5%), followed by grade 3 leak (*n* = 30, 34.5%) and grade 1 (*n* = 20, 23%). The histological diagnosis and respective intraoperative grades are summarized in Table 2. Exemplary cases are demonstrated in Fig. 1.

**Table 1:** Classification of intraoperative CSF leakage in the 87 (of 280) patients with intraoperative identified CSF flow

grade by Esposito	number of patients	percentage
grade 1	20	23%
grade 2	37	42.5%
grade 3	30	34.5%

**Table 2:** Histological diagnosis and respective intraoperative CSF fistula grade (*n* = 87)

	Intraoperative CSF fistula grade 1	Intraoperative CSF fistula grade 2	Intraoperative CSF fistula grade 3	Total
Adenoma	19	36	1	56
Meningioma			8	8
Rathke´s cleft cyst	1		5	6
Craniopharyngioma			6	6
Chordoma		1	3	4
Other pathologies			7	7
Total	**20**	**37**	**30**

### Surgical treatment of intraoperative CSF flow and postoperative outcome

**Fig. 2:**
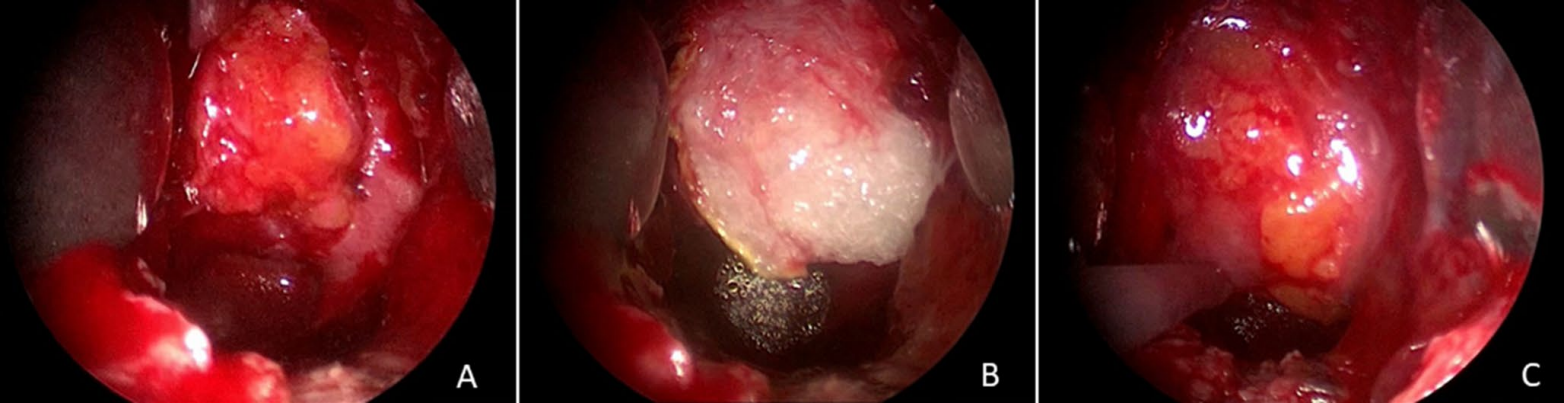
Exemplary case of closure of a CSF leak grade 2 with intradural/intrasellar autologous fat graft (**A**), Tachosil (**B**) and additional autologous fat graft (**C**) as outer layer in sphenoid sinus

The materials commonly used for the closure of grade 1 CSF leaks were primarily sealant sponge Tachosil ^®^ (70%), fibrin glue (55%) and bone (55%). For grade 2 CSF leaks, Tachosil ^®^ (78%), bone (57%) and autologous fat (54%), here the fibrin glue is less routinely used. In the grade 3 CSF leaks, the combination of lumbar drains (90%), autologous fat graft (87%) and fascia lata (60%) constitutes the primary strategy for the closure (see Table 3). A total of 63 patients received an intraoperative lumbar drain (72%). There was no significant association between the used closure material and the developing postoperative CSF fistula, for bone (*p* = 0.598), autologous fat graft (*p* = 0.153), fascia lata (*p* = 0.625), sealant sponge (TachoSil^®^) (*p* = 0.688), fibrin glue (*p* = 0.09), and intraoperative use of lumbar drains (*p* = 0.751). Figure 2 shows a closure in sandwich-technique with autologous fat graft intradural/intrasellar, Tachosil and additional fat graft in sphenoid sinus.

**Table 3:** Materials used in the different grades (according to Esposito et al.) of intraoperative CSF leakage

Intraoperative CSF leakage	Autologous fat	Fascia lata	Sealant sponge (Tachosil ^®^)	Fibrin glue	Bone	Additional lumbar drainage
grade 1 (*n* = 20)	2 (10%)	1 (5%)	14 (70%)	11 (55%)	11(55%)	4 (20%)
grade 2 (*n* = 37)	20 (54%)	4 (11%)	29 (78%)	17 (46%)	21 (57%)	32 (86%)
grade 3 (*n* = 30)	26 (87%)	18 (60%)	20 (67%)	7 (23%)	14 (47%)	27 (90%)
Total (*n* = 87)	48 (55%)	23 (26%)	63 (72%)	35 (40%)	46 (53%)	63 (72%)

### Postoperative CSF fistula

None of the patients without intraoperative detected CSF (Esposito grade 0) of the whole cohort of 280 retrospectively analysed cases developed postoperative a new CSF fistula. From the total of 280 (including the patients 87 patients with intraoperative CSF leak and 193 without CSF leak), only 9 cases of persisting postoperative CSF leakage were identified. Making a total of 3.2% of postoperative CSF leakage in the presented cohort. The details of these 9 patients are demonstrated in Table 4. All the nine postoperative CSF leaks were observed in the patients with intraoperative CSF leak, which corresponds to 10% (9/87) of the patients with intraoperative identified CSF flow. There was no significant difference in the probability of a new postoperative CSF fistula according to the intraoperative grading of CSF flow: 10% (*n* = 2) of all grade 1 leaks, 10.8% (*n* = 4) of all grade 2 leaks and 10% (*n* = 3) of all grade 3 leaks developed a postoperative CSF leak. This implies that within each CSF leakage grade approximately 10% will experience failure of the intraoperative closure technique.

**Table 4:** Characteristics of patients with new postoperative CSF leakage in the presented series with detailed illustration of used closure material

Case	Pathology	Grading of leakage	closure material
case 1	adenoma	grade 1	Bone, Tachosil, fibrin glue
case 2	adenoma	grade 1	Autologous fat, bone
case 3	adenoma	grade 2	Autologous fat, bone, Tachosil
case 4	adenoma	grade 2	Autologous fat, bone, Tachosil, fibrin glue
case 5	adenoma	grade 2	Autologous fat, Tachosil, fibrin glue
case 6	meningioma	grade 3	Autologous fat, fibrin glue, fascia lata
case 7	chordoma	grade 3	Autologous fat, fascia lata
case 8	arachnoid cyst	grade 3	Autologous fat, Tachosil, fibrin glue, fascia lata
case 9	rathke cleft cyst	grade 2	Bone, Tachosil, fibrin glue

Overall these results indicate that patients with intraoperative CSF flow have a significantly higher risk of developing postoperative persisting CSF fistulas compared to patients without intraoperative CSF leakage (*p* < 0.001). In our cohort, the grade 2 and grade 3 classified intraoperative CSF leakages (44.4% and 33.3%) were more prone to develop a postoperative persisting CSF fistula compared to the grade 1 cohort (22.2%) but statistical no significant (*p* > 0.05).

A meningitis was detected postoperatively in six patients, of which five also had a postoperative CSF fistula and were treated with lumbar drains. Only one patient with meningitis has never had a lumbar drain.

All of the nine patients with a persisting postoperative CSF fistula, were treated with a lumbar drain and bed rest for at least 5 days. In only three cases (33.3%), the use of a lumbar drain was successful. The other six patients (66.6%) with persistent postoperative CSF fistulas had to undergo revision surgery with revision of the dural reconstruction. All of them were treated successfully and presented with no complications.

During follow-up, no further complications, no new CSF fistula was detected.

## Discussion

Postoperative CSF fistula is a potential complication that can occur following endonasal endoscopic skull base surgery. This procedure, which allows access to complex areas of the skull base through the nasal cavity, has become increasingly popular due to its minimally invasive nature and favorable outcomes. However, the risk of developing a CSF fistula remains a concern. Different surgical techniques and closure methods have been introduced in skull base surgery. Thereby, the probability of postoperative CSF fistula should be decrease up to less than 5% in complex cases nowadays.

The use of the Valsalva maneuver is very helpful in testing the absence of intraoperative CSF leak that could otherwise be overlooked. Esposito proposed a protocol for sellar repair as follows: In grade 0, only collagen sponge is used. In grade 1 leaks, a single layer of collagen sponge is placed over the exposed pituitary gland, followed by a titanium mesh buttress wedged into the intrasellar, extradural space, followed by a second layer of collagen sponge placed over the mesh. In grade 2 leaks, intrasellar abdominal fat graft and collagen sponge, followed by titanium mesh buttress and additional fat. For grade 3 leaks, the same technique as for grade 2 leaks and use of a lumbar drain for 48 h are used. With this technique, leak repair failures have been described in 2,5%, where grade 3 leak failure rates being the most common [17]. Zador has described another protocol for repairing the sellar floor, according to the classification made by Esposito [21]. In grade 0, only a hemostatic gelatin sponge is placed in the tumor bed. In the grade 1 leaks, a hemostatic gelatin sponge and fibrin glue (for example DuraSeal, BioGlue) can be used. In grade 2 leaks, an additional autologous fat graft is used. For grade 3 leaks, the defect is closed with fascia lata graft, autologous fat and/or gelatin sponge and a vascularized nasoseptal flap [21].

This presented study analyzes the outcomes of the closure presented multilayer technique applied adapted to the intraoperative grade of CSF fistula via endoscopic transsphenoidal approach. The results should be correlated to other well established techniques as e.g. the pedicled neurovascular flap. We demonstrated a probability of CSF fistula postoperatively of 3.2% overall. This is in line with previous publications and not inferior to results which have been described using a nasoseptal flap in these cases [9, 14, 36].

Analyzing only the patients with intraoperatively identified CSF leakages, the probability of CSF fistula is higher with 10%. This underlines the effectiveness of detecting an intraoperative CSF fistula by an experienced neurosurgeon with the endoscope. At this point, we suggest that the Valsalva maneuver plays an important role in the detection of the small leaks, leading to an optimal closure and treatment of the intraoperative CSF grade 1 leaks. Based in this surgical technique, we detected every CSF flow intraoperatively, even if it was only a minimal flow.

This rate indicates that patients with intraoperative CSF flow have a significantly higher risk of developing postoperative persisting CSF fistulas compared to patients without intraoperative CSF leakage. Additionally, we will see more closure technique failures in grade 2 and 3 patients and that in these grades a meticulous attention should be paid to the reconstruction of the dural defect.

The use of different types of material for the closure was analyzed and we found no association for a better outcome in correlation to a specific material. However, the use of autologous fat graft and fascia was significantly higher in the cohort of grade 2 and 3 leakages intraoperatively. Additionally, the use of lumbar drains was significantly higher in the patients with intraoperative grade 2 and 3.

Our analysis revealed that none of the materials was associated with an increase or decrease in the risk of a persisting postoperative CSF fistula. We postulate that the experience of the surgeon is of great importance to the surgical outcome, although this fact cannot be analyzed objectively in this setting.

The grading of CSF leak intraoperatively showed no association in the development of postoperative persisting CSF fistulas. That demonstrates that bigger intraoperative CSF leaks, are not necessarily related to higher risks of postoperative CSF fistulas. That could be explained by the different measures taken for the closure techniques in the different grades of CSF leak. The higher the grade of intraoperative CSF leakage, the more intensive and complex was the reconstruction technique. In the grade 1 group, most patients were treated mostly with TachoSil and fibrin glue, whereas in the grade 2 the closure was performed with Tachosil and beginning the use of fat graft and lumbar drains, and in the grade 3, almost all patients had an autologous fat graft and lumbar drains.

In order to evaluate the efficacy of our technique, we compared our cohort with other results in the literature. Thereby, the results revealed comparable results with similar rates of persistent postoperative CSF fistulas with nasoseptal pedicled flap and other techniques. Several studies have observed an incidence of postoperative CSF fistulas, for example Conger et al. reported a significantly lower leak rate of 1.6% [40], and Kuan et al. [41] reported a rate of 2.3%. However Chen et al. presented a higher rate of postoperative CSF fistulas in 8.4% with a multilayer technique [5, 12, 13, 16, 30]. Shahein et al. reported postoperative CSF leaks from 0 to 6% with the use of collagen matrix and mucoperiosteum graft respectively [29]. Other papers with similar CSF leak rates to ours have been reported by Amano et al. [42] and Hara et al. [43]. who used pedicled nasoseptal flaps selectively for Grade 3 intraoperative CSF leak cases.

The better result of our analysis might be induced by the consequent use of a lumbar drainage [20, 24]. However, we will analyze the benefit of lumbar drains in further studies.

Furthermore, many authors did not analyze and correlate their final numbers of postoperative CSF fistulas to the intraoperative identified probability of CSF flow and the grade of the CSF leakage intraoperatively. This intraoperative identification and analysis by the neurosurgeon are essential for the use of the best closure technique and the postoperative result.

In some studies, the postoperative CSF fistula were statistically higher in patients with meningiomas [17].

In the presented patient cohort, the pathology of the tumor as well as tumor size was not associated with a significant risk for the development of a postoperative CSF fistula. Meningiomas, craniopharyngeomas and chordomas were associated to higher grades of intraoperative CSF leak but not to an increased risk of developing new postoperative CSF leaks. This could be explained by the fact that the surgeon would expect a CSF leak in these intradural or invading tumors, since they involve the dura and are represent larger tumors per se in the sella region. Due to that fact, the surgeon is more careful and performs a more meticulous and precise closure in those types of pathologies. According to this study, tumor volume is not related to an increased risk of developing a new postoperative CSF leakage. This result is because the size of the tumor is not necessarily associated to the size of the diaphragmatic defect intraoperatively. Additionally, these tumors were not removed en bloc normally by the neurosurgeons. The typical surgical technique is a debulking and piecemeal technique.

On the contrary, according to other studies that confirm that the rate of postoperative CSF leakage is lower in pathologies without dural involvement, such as pituitary adenomas [14], we did not observe lower postoperative CSF leaks in the pituitary adenomas. This could be explained by the same argument as above. The surgeon, not anticipating a leak in these pathologies, might have underestimated the adenomas and the extension of the pathology, meaning that the closure was not as meticulous and careful as in the other pathologies such as meningiomas or chordomas.

The only patient with an arachnoid cyst in the cohort developed also a new postoperative CSF leakage. Like described before, this type of pathology involves an intra arachnoid lesion that is related to a directly intraoperative high flow CSF fistula. Especially the high flow CSF leak in this case may induce an increased risk of developing a postoperative CSF leakage. However, we can assume this aspect because only one patient presented this type of pathology.

This study has some limitations. We included various intrasellar pathologies—such as adenomas, meningiomas, Rathke cleft cysts, and others—which may complicate achieving statistical significance. For a more robust analysis, a prospective study with a more homogeneous group of pathologies is recommended, involving a larger number of cases and studies.

## Conclusion

We suggest establishing a standard closure procedure with a standardized use of same materials in the transsphenoidal endoscopic surgery according to the identified grade of intraoperative CSF fistula, such as all cases with intraoperative CSF grade 1 should be reconstructed with fibrin glue, autologous bone and Tachosil. All intraoperative CSF fistulas grade 2 should be reconstructed with the same materials as in grade 1 cases with additional autologous fat graft. And all intraoperative CSF fistulas grade 3 should be reconstructed as before with the additional use of facia lata as an underlay technique intradurally. Probably, the combination with lumbar drainage could be even more effective and should be analysed im detail, additionally with another aspect in future work.

The novelty of this study lies in the detailed analysis of postoperative CSF leak rates stratified by intraoperative CSF leak grade, which has not been thoroughly investigated in prior reports.

As the authors acknowledge, the favorable outcomes are likely attributed to the precise intraoperative assessment and individualized closure strategies based on accumulated surgical experience. However, this detailed approach, particularly the systematic identification of intraoperative CSF leakage and decision-making for closure technique provides valuable insights for the next generation of skull base surgeons.

We can conclude that the presented multilayer technique might offer a safe, effective alternative technique for the closure and reconstruction of the dura and sellar floor after endoscopic transsphenoidal surgery. The presented surgical techniques offers an alternative to the pedicled nasoseptal flap with less nasal surgical complications because of less invasive surgical steps in the nostril. However, this decision-making of the better closure technique should remain flexible, allowing the surgeons to choose the appropriate technique according to surgeons experience, need and benefit for each individual case. As an important resource, the Valsalva maneuver should be applied in all cases to detect even very small defects of the diaphragm and minimal cerebrospinal fluid (CSF) flow intraoperatively.

Future studies with larger cohorts and standardized reconstruction protocols for each CSF leak grade may help establish a less invasive, flapless closure strategy as a reliable alternative and serve as an important educational reference. Another aspect to take into consideration for the next studies should be the sellar barrier, which can suggest a higher risk of intraoperative CSF leak [44] and consequently prepare the surgeon for a better closure decision-making – probably even by a presurgical evaluation of the MR images.

## Supplementary Information

Below is the link to the electronic supplementary material.

Supplementary File 1(MP4 18.3 MB) Exemplary case of closure in CSF leak grade 1 with autologous fat graft and Tachosil collagen

Supplementary File 2(MP4 60.8 MB) Exemplary case of closure in CSF leak grade 3 with autologous fat graft, facia lata and DuraSeal glue

## Data Availability

No datasets were generated or analysed during the current study.
